# Exploring the Experiences of Family Members When a Patient Is Admitted to the ICU with a Severe Traumatic Brain Injury: A Scoping Review

**DOI:** 10.3390/jcm12134197

**Published:** 2023-06-21

**Authors:** Kati Hayes, Sam Harding, Kirsten Buckley, Bronagh Blackwood, Jos M. Latour

**Affiliations:** 1 Research and Development Department, North Bristol NHS Trust, Westbury on Trym, Bristol BS10 5NB, UK; sharding.jb@gmail.com; 2 Learning and Research Centre, North Bristol NHS Trust, Westbury on Trym, Bristol BS10 5NB, UK; kirsten.buckley@nbt.nhs.uk; 3 The Wellcome-Wolfson Institute for Experimental Medicine, Queens University Belfast, Belfast BT7 1NN, UK; b.blackwood@qub.ac.uk; 4 School of Nursing and Midwifery, University of Plymouth, Drake Circus, Plymouth PL4 8AA, UK; jos.latour@plymouth.ac.uk; 5 School of Nursing, Midwifery and Paramedicine, Curtin University, GPO Box U1987, Perth 6845, Australia

**Keywords:** severe traumatic brain injury, family members, caregivers, intensive care, critical care, scoping review

## Abstract

The needs of family members of patients in the intensive care unit (ICU) with a severe traumatic brain injury (TBI) remain unmet. To date, no review has been performed to explore the experiences of relatives of adults who have been admitted to the ICU for treatment of a TBI. The aim of this scoping review is to explore and map the evidence of the experiences of family members when an adult relative is admitted to an ICU with a severe TBI. This review follows a combination of guidelines from Arksey and O’Malley and the Joanna Briggs Institute. Five electronic databases, Medline, Emcare, Embase, CINAHL, and PsycInfo were searched in February 2023, as were a number of grey literature sources. The population, concepts, and context framework were used to define the inclusion and exclusion criteria. From 4077 records, nine studies were retained, which represented seven discrete studies. The experiences of family members were thematically analyzed. The narrative synthesis of findings revealed three themes: communication with the clinical team, uncertainty, and involvement in care. These results offer richness and depth of understanding to clinicians regarding the experiences of families during this traumatic time. This review provides direction for targeted interventions aimed at supporting family members while in the ICU.

## 1. Introduction

### 1.1. Background

Traumatic Brain Injuries are a leading cause of death and disability worldwide [1,2,3,4]. The World Health Organisation (WHO) identifies Traumatic Brain Injury (TBI) as a global health concern [1]. Younger people are most affected by a TBI, with a majority of those injured being under 40 years of age [4,5].

Traumatic brain injuries can be classified as mild (Glasgow Coma Scale (GCS) score 14–15), moderate (GCS 9–13), who may or may not require care in the intensive care unit (ICU), and severe (GCS 3–8) who require invasive ICU treatment. Patients with severe TBI injuries can be extremely unstable, requiring immediate life-saving measures, which often include emergency surgery [6,7].

For family members, this acute period in ICU can be distressing, with high levels of stress and anxiety reported [2,8,9]. In the early stages, patients in the ICU who have had a severe TBI are unconscious and require assisted ventilation. This adds a level of uncertainty that is unique to the ICU environment [9,10]. The ICU also poses a threat to the equilibrium of the family system by separating the patient from the family members [9], with the structure and functioning of a family being profoundly impacted [7,8].

### 1.2. Rationale

The long-term effects on family members living with a loved one who has a severe TBI have been previously investigated. However, to date, the experiences of family members of patients who have been in the ICU have not been reviewed. It appears that the level of post-traumatic stress experienced following the ICU phase at three months is higher amongst family members of a patient with a TBI than without a TBI [2,9]. In the longer term, elevated levels of stress and depression have been found among family members of TBI survivors who are caregivers, which can lead to adverse psychological and psychophysiological damage [3,7,9], with caregivers often requiring professional intervention [8]. This evidence suggests sequelae of psychological impact and the need for support specific to this family group beginning in the ICU. Coco et al. completed a systematic review of the support for TBI patients’ family members in neurosurgical nursing [4]. However, the review did not focus on the ICU family experience. Similarly, Whiffin et al. carried out a meta-synthesis of narrative structures of the experience of families following a TBI in adults that looked at the sub/post-acute period but not the acute phase in the ICU [5]. Weitzig et al. completed an integrative review of the needs of families of ICU trauma patients. Still, it did not identify specific needs relating to those of ICU trauma patients with a TBI [8]. To date, no other systematic or scoping reviews have been identified that related to family members’ experiences when a patient with TBI is admitted to an ICU.

The purpose of the current review is to explore and map the experiences of family members when an adult is admitted to an ICU with a severe TBI. The review will provide a synthesis of current evidence that will inform healthcare professionals caring for patients with a severe TBI in the ICU. A scoping review approach has been adopted as scoping reviews are useful in exploring and mapping concepts in a research area [11]. This exploration allows for a broad overview while not being focused on asking what works. It presents findings irrespective of study quality to clarify key concepts and to identify gaps in the evidence [12].

### 1.3. Research Question and Objectives

The scoping review question was: What are the experiences of family members when an adult relative with a severe TBI is admitted to the ICU? The objectives of this review were (1) to investigate the experiences of family members when an adult with severe TBI is admitted to an ICU and (2) to develop a narrative meta-synthesis of the themes from qualitative data of the included articles.

This scoping review follows a combination of guidelines from Arksey and O’Malley [11] and the Joanna Briggs Institute (JBI) for conducting and reporting scoping reviews [12]. Arksey and O’Malley’s framework offers a six-step systematic approach to map the literature in the area of interest [11]. These steps include (1) formulating the review question, (2) identifying the relevant literature, (3) selecting studies, (4) mapping out the data, (5) summarizing, synthesizing, and reporting the results, and (6) including expert consultation. Combining this approach with the JBI framework allows the use of the Population, Context, Concept (PCC) framework outlined by the JBI guideline.

## 2. Materials and Methods

### 2.1. Protocol and Registration

An *a priori* protocol was developed, and the study was registered with the Open Science Framework in December 2021 (https://doi.org/10.17605/OSF.IO/YZEPM, accessed on 20 June 2023). Searches were undertaken in February 2023. The results of the scoping review are reported using the reporting guideline PRISMA Extension for Scoping Reviews [13] (Electronic Supplementary Materials 1).

Using the PCC framework, the research question was divided into three key concepts: Family members of patients with TBI, patients with TBI in the ICU, and experiences of family members. The search strategy was developed, piloted, and refined with the support of a knowledge information specialist (clinical librarian). The search terms for traumatic brain injuries were included using terms outlined by the Cochrane Library [14]. The final search strategy is presented in Electronic Supplement Materials (ESM) 2.

### 2.2. Eligibility Criteria

Key inclusion and exclusion criteria were defined (Table 1).

### 2.3. Information Sources

Five databases were searched: CINAHL, Embase, Emcare, Ovid Medline, and PsychInfo. Only English texts were included due to study resources; however, a date limit was not set to allow for the inclusion of historical research that may be relevant to the area of interest. The search was conducted from inception to 1 February 2023.

Papers could include but were not exclusive to self-reported family experiences, reports by health care providers, or former patients of the ICU. Reviews and opinion articles were excluded as these articles would not include primary data related to the experiences of the population.

The grey literature was searched using the national grey literature collection (https://allcatsrgrey.org.uk, accessed on 10 February 2023). To ensure that pre-printed research is included in the grey literature search, pre-printed servers medRxiv (www.medrxiv.org, accessed on 10 February 2023) and PsyArXiv (https://psyarxiv.com, accessed on 10 February 2023) were searched. A google scholar search was undertaken, with the first ten pages of returns reviewed for inclusion. These were searched using the EThOS (https://ethos.bl.uk, accessed on 10 February 2023). The reference lists for any retained papers were searched for additional papers.

### 2.4. Search Strategy

Criteria for inclusion and exclusion were set a priori (Table 1), forming the basis of the search strategy. A copy of the full strategy for the Ovid Medline search is provided in Electronic Supplement Materials 2.

### 2.5. Selection of Sources of Evidence

**Figure 1:**
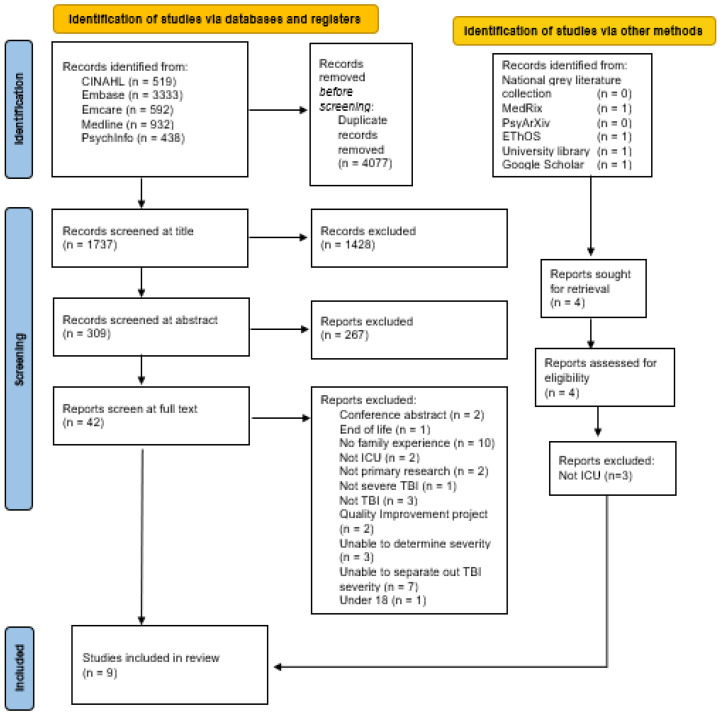
PRISMA ScR Flow chart [17].

All identified citations were collated and uploaded into Endnote [15], and duplicates were removed. The deduplicated references were uploaded into the Rayyan reference management database [16], allowing for a blinded assessment of the papers between the authors. Papers were screened at title level, then by abstract. Two reviewers independently reviewed all abstracts according to the criteria in PCC (Figure 1) at both of these stages. At the full-text stage, inclusion criteria and exclusion criteria were discussed with the second author and refined. This process was undertaken as it was recognized that the population terminology “family member” was only evident in full text in several search results. The level of agreement between the two reviewers was 100%. Following the abstract review, full texts were independently reviewed, and reasons for the exclusion at the full-text level are present in Electronic Supplementary Materials 3.

### 2.6. Data Charting Process

Once the final included papers were agreed upon, data extraction was undertaken by the authors KH and reviewed by SH. This data included patient and family member demographics, as well as themes and quotes derived from the individual pieces of research. The PRISMA-ScR flow chart (Figure 1) shows the number of references under consideration at each stage.

### 2.7. Critical Appraisal

Findings were presented but not ‘weighted’ in terms of quality. Although, following the Arksey and O’Malley guidance, a quality appraisal was undertaken [11]. The quality appraisal tools were selected from the Critical Appraisal Skills Programme (CASP) family of tools, depending on the study design of the individual retained articles [18].

### 2.8. Synthesis of Results

A narrative synthesis approach was chosen to synthesize the diverse range of selected studies in a structured manner, following the European Social Research Council Guidance on the Conduct of Narrative Synthesis in Systematic Reviews [19].

## 3. Results

### 3.1. Selection of Sources of Evidence

In this scoping review of the literature, 4077 records were returned once duplicates were removed. One thousand seven hundred thirty-seven records were screened for title and abstract, 42 articles underwent full-text review, and nine publications representing seven primary research studies [10,20,21,22,23,24,25,26,27] met the inclusion criteria (Table 1).

### 3.2. Characteristics of Sources of Evidence

Table 2 provides an overview of the demographics and PCC elements of each of the retained studies. Countries represented in these articles are the USA (n = 3) [20,26,27], Canada (n = 3) [10,22,23], Scotland (n = 1) [25], India (n = 1) [21] and Thailand (n = 1) [24]. Methodologies reported include qualitative interviews (n = 8) and one quantitative descriptive study.

The experiences of 148 family members are represented across the seven studies. Of 148 participants, 48.6% (n = 72) are male, and 51.4% (n = 76) are female. The method of reporting participants’ relationship to the patient differs across the studies, for example, being described as parent, spouse, or sibling [21,22,23], while more specific relationships are outlined in other studies retrieved, such as mother, father, sister, wife [20,24,25,26]. In other papers, gender only is reported [22,23]. The terminologies “Surrogate decision-maker” and “caregiver” were used in three separate studies [21,26,27]. However, it was evident from the full text that this terminology referred to family members. Patients were identified as having severe traumatic brain injuries by the degree of severity outlined in the title, abstract or full text. The severe nature of the injury was inferred from the terminologies “critically ill in ICU” or “critically ill with TBI” in three studies [25,26,27], while it was overtly stated as “severe” in the majority of articles [10,20,21,22,23,24].

The context of the study is clearly stated as being ICU in most investigations [10,21,21,24,25] and is inferred as being ICU from “level 1” and “critically ill patients” in two studies [26,27]. One investigation reported in two papers involved participants who were two years or more after the initial injury [22,23], and another contained two time points of the patients’ journey [10]. In these instances, data was extracted solely from the ICU stay.

### 3.3. Methodological Approaches

Interviews were predominantly the method of data collection in the studies identified. Two primary studies using semi-structured interviews with broad, open-ended questions [22,26] were followed up with secondary analyses of the content and included as separate published studies in this review [23,27], with only one [23] declaring that the paper was a secondary analysis. Semi-structured interviews, with thematic analysis of the data, were adopted by one study that formed the qualitative element of a larger mixed methods study [10]. Two studies employed an exploratory, descriptive design approach [20,24]. Grounded theory methodology was adopted to gain an understanding of a family systems approach by one researcher, using group interviews for data collection [25]. Finally, one quantitative study was included with depression, anxiety, and stress scale (DAS-21) questionnaires administered to participants [21]. Qualitative data from the retained papers has been thematically analyzed, and the authors present this in the next section.

### 3.4. Critical Appraisal within Sources of Evidence

CASP (2021) does not recommend that studies are scored but rather rated as ‘low’, ‘medium’, or ‘high’ [18]. 22.2% were high quality (n = 2), 44.4%, medium quality (n = 4) and 33.3% low quality (n = 3) [Electronic Supplement Materials 4].

### 3.5. Synthesis of Results

Table 3 provides the aims stated in the nine included papers and the original themes reported by the authors. Qualitative data from the papers were thematically analyzed, and a narrative synthesis of the findings is reported below with three themes: (1) “Communication with the clinical team”, (2) “Uncertainty”, and (3) “Involvement in care”, being derived from the authors of the current paper.

### 3.6. Narrative Synthesis

#### 3.6.1. Communication with Clinical Team

From the studies retained, it is evident that the families of patients in the ICU with a TBI are impacted by how clinical teams communicate with them. Inconsistency in communication has been highlighted as causing distress to family members [20,22,26]. Frustration is reported because of being given differing advice from different members of the professional team and doubting if they are being told the truth [22]. Families found that receiving conflicting information was harmful to them, expressing that “each doctor said something different… it was very confusing and problematic; we almost started fighting” [20]. Families also found that a lack of cohesiveness in communication from the clinical team made decision-making about goals of care difficult to make [27].

As the patients’ journey progressed in critical care, the emotional distress experienced by the family was compounded by this unmet need for consistent communication [20]. One study describes this need for communication as being an “intense need” [10]. Clear, understandable language is needed, with families reporting experiencing that medical “jargon” leads to a lack of understanding/misunderstandings [20,27]. In addition, a sense of ambiguity or vagueness in communication left families feeling ignored and added to the difficulties that they have adjusted to in their lives [23]. A counterpoint to this was presented in one study where participants voiced a strong desire for more ambiguity in communication regarding the prognosis of the patient and felt that more ambiguity might bring more hope into these conversations [27].

Overall, facts were sought by families and not false hope. A desire for honesty and prompt communication from the start of a patient’s journey was expressed by families [20,26]. Positive experiences with health professionals are achieved when families feel listened to [22], with one study highlighting that strong support is felt by family members when communication is upfront, forthcoming, and honest [10]. Families experienced that while the communication experience with professionals was directly supportive, it was also guiding them on how to communicate with their wider communities and helped to navigate interactions with wider families [10].

#### 3.6.2. Uncertainty

Families experience profound uncertainty during a patient’s time in ICU due to having a severe TBI. Subthemes of uncertainty in decision-making, uncertainty in prognosis, and existential uncertainty emerged during the analysis.

##### Uncertainty in Decision Making

A sense of frustration is evident when this experience of uncertainty in decision-making is described [28]. The sentiment of uncertainty in their own decision-making is something that families felt unprepared for, struggling for many months afterward [26,27]. One participant said, “Uncertainty makes it [decision making] the hardest part” [26]. Families report that they lack support in addressing their uncertainty and that sensing uncertainty from health professionals causes doubt; “In my opinion, after seeing them for a long time, they didn’t seem to know very much” [22].

Uncertainty around clinical goals of care was felt by participants. A perceived lack of cohesiveness within the clinical team regarding decision-making was emotionally distressing for family members. One participant reported that “the nurse made it clear that his [the nurse] views were not the same as the doctors… that caused us a lot of additional stress, anxiety, and second-guessing. It was a whole night of torture” [26]. This sense of uncertainty crosses over with a need for cohesive communication within the clinical team previously discussed, resulting in mistrust among family members in several studies [22,23,26,27].

##### Uncertainty in Prognosis

Uncertainty surrounding prognosis is experienced by families [10,22,23,24,25,26,27]. One participant stated, “he is so critical…he was hour by hour, you know; he was like that for days, and that would be the hardest…the waiting and the unknown” [10]. This sense of certainty or guidance through the process of predicting the prognosis is sought by family members through numeric predictions [26]. 82% of participants preferred to receive exact numeric estimates from the clinical team when discussing prognosis, finding it was “more clear, more concise, it less confusing; there is one statement made” [26]. In contrast to this, other families did not want statistics or probability. They wanted specific information only about their loved ones [10].

It’s clear that for a family’s prognostic uncertainty is not just expressed as survival. Families experience uncertainty about how life will be and change due to the injuries sustained [10,25]. Families seek to “map the future” while the patient is still in ICU, with the impact of the injury on individual family members’ life described as an “elusive concept” [25]. With family members describing their loved ones, TBI as having “changed my life. It changed our life” [25].

##### Existential Uncertainty

Uncertainty about the existential presence of a loved one in the ICU is apparent through the experience of ambiguous loss in families [25]. The physical presence and psychological absence of the patient in the ICU were described as “All they can tell you is that there’s electrical activity and body functioning. It’s like he’s not really there… it’s like his body is there, but it’s really hard to see that he is there… nothing comes forward” [25]. Uncertainty or ambiguity of the loss of the person is expressed in this study also where participants are described as referring to their loved ones in the past tense, for example “he had the world at his feet” [25].

#### 3.6.3. Involvement in Care

The theme of the experience of ‘involvement in the care’ of a loved one in the ICU with a TBI was found in the studies reviewed. Two subthemes were identified; Family involvement in ICU care and future involvement after ICU, as families experience internal planning and preparation for the next stage of life with a loved one with a TBI.

##### Family Involvement in ICU Care

Family members express their need to be physically close to the patient, and the involvement with the patient’s care translates as being present in tactile elements of caring [10]. Involvement is a positive experience for some families when they are recognized for their knowledge of the patient, and their involvement is to “help them understand who [the patient] is” [22]. However, it is felt as damaging when the recognition of the families’ presence is not given in ICU. The experience of feeling “brushed aside [for the family] was really hard to take” [19,22].

Direct involvement in care was a means of managing the challenges that they, the family members, faced in the ICU. When describing physical care, they felt that “these actions will help him feel comfortable and stimulate his consciousness” [24].

A sense of responsibility was evident in the interview responses; “I need to stop working on the farm in order to visit and take care of my son”; “I have to wake up about 4 a.m. and take the bus to the hospital” [24]. Other family members describe how they relocate to temporarily live near the hospital or sleep in the waiting room to be present early to resume caring duties [24].

Changes needed within the wider family are alluded to, with reference to the husband of a participant taking over household duties [24]. When describing physical care, a participant felt that “these actions will help him feel comfortable and stimulate his consciousness” [24] and are described as a means of managing challenges by the family. Conversely, in a quantitative study with 75% male participants, the caregiving role was associated with higher levels of stress and anxiety than their female counterparts in the study, and a sense of the burden of care was felt [21].

##### Future Involvement after ICU

Families experience a shift in the structure and functioning of a family when a patient has a severe TBI in the ICU. Described as “a dividing line between before and after, the instant when the life of a person and their family is turned upside down” [23]. A participant describes this shift in terms of “That was when it all began…or ended…I don’t know” [23].

This sense of transition is highlighted in a study in ICU amongst families in which “mapping the future” was highlighted as a major theme [25]. Mapping the future is described as how a person refocuses their “time perspective from present to future” [25], here being the emerging caring scenario that families are facing while in the ICU and the implications on different family members. Families were mentally preparing themselves for the women to be the future caregivers. Women interviewed conveyed a sense of needing to “give up” work to care for the patient [25]. One mother says that “they [daughter and husband] can carry on with their lives, but I can’t because of him [the patient]” [25], and another participant recounts that she can no longer make plans for leisure or work. The future is described as “Future? What future?” [25].

Fear of the involvement of future care that will be needed was evident. However, a willingness to learn how to care for patients was also voiced. The acceptance and of a need for future involvement in care appeared evident, saying that she “would maybe have to do these things for him a bit later down the line… I’m as well finding out now” [25].

## 4. Discussion

This review aimed to explore the experiences of family members when a loved one is critically ill in the ICU with a severe TBI and to provide a narrative of how family members experience this difficult time. The themes uncovered first highlight the importance of communication with the clinical team. Secondly, the theme of uncertainty encompassed three subthemes of uncertainty in prognosis, decision-making, and existential uncertainty of the loved one in the ICU. Finally, family members’ involvement in patient care incorporated subthemes of involvement in care in the ICU, and future involvement was explored as families experience internal planning and preparation for the next stage of life with a loved one with a TBI.

The clinical condition of patients with severe TBIs can change rapidly. This may influence the consistency of communication with family members. This review highlights that the lack of consistent, clear, and honest communication with the clinical team may increase the psychological burden of family members while in the ICU. This echoes the findings by Coco et al., who sought to describe what constitutes support for the families of patients with a TBI [4]. Families were found to trust the information that was given to them more when it was consistent with the information given by their peers [4]. Although the review by Coco et al. is not based uniquely on the ICU experience, similar needs were found regarding honesty in communication. Families feared that they were not communicated with truthfully on occasion and felt that they needed to seek medical information from the clinical team, leading to frustration and mistrust [4]. The current review also highlights that mistrust can develop in family members when information is not readily available and forthcoming.

Coco et al. identify that emotional support is shown by caring, listening, and respecting the family in times of communication [4]. Families in this current review recognize the moments of being listened to as positive experiences with the clinical team [10,22]. Memories of when communication was difficult, or families felt unsupported are painful for families in the retrospective recall, with memories lasting for years after the experience [23]. The timing of receiving the information has been raised by previous research as an important aspect in maintaining clear communication of information. If information is given early into an acute stay for a patient with a TBI, families have experience that they are unable to internalize the information received [4]. However, the current review highlights a desire for prompt and timely communication from medical teams [19,26]. Further investigation may be needed to identify clinician awareness of the importance of the softer and more nuanced elements of communication and how best to ensure that families are supported in receiving and, importantly, retaining complex and often distressing information.

The way in which uncertainty is experienced by families in the ICU is multifaceted. Families appear to seek to feel grounded in decision-making regarding goals of care, with lasting detrimental impacts when uncertainty is experienced. Family members express painful repercussions when they are unsupported in their uncertainty, which highlights perhaps a lack of awareness by the clinical team of how vulnerable families are at this time of a patient’s journey in the ICU. However, families also need to be supported in preparing for ongoing uncertainty. Whiffin et al. describe a post-acute phase of living with a TBI as the “rupturing” of a family unit’s narrative, with families “fighting to keep a foothold” to create a new stable narrative [5]. This process of living with uncertainty begins in the ICU.

Prognostic uncertainty is clearly expressed as struggling with the unknown [10]. There is a sense of a natural desire to prepare for what is to come sought by family members. This was found by Coco et al., who found that families needed to understand the prognosis to be prepared to cope at home after the hospital admission [4]. However, while clinical teams can offer numeric probabilities that may be helpful to some families, caution is needed as clinicians frequently cannot provide certainty in the early stages of injury and experience the same uncertainty as families [22,23]. In addition, clinicians ought to be aware that family members can find reassurance and hope in prognostic ambiguity, with a balance needed so that false hope is not raised.

Families can feel that they have lost the person that they once knew and loved, and while they are physically present, families feel uncertain of whether the essence of their loved one is there [20]. There may be steps of grief and mourning that lie ahead of the families of patients in the ICU with a severe TBI. Families in the post-acute period can feel that the brain-injured person is a “new person” [5]. Whiffin et al. describe this as processing unresolved grief. With this considered, further research may be needed to establish whether families of patients with severe TBIs may be specifically at risk of developing complex or prolonged grief difficulties in the future [5].

The current review reveals how experiences of involvement in care differ according to cultural expectations. Families in Eastern countries [21,24] appear to take a greater “hands-on” approach to the care of a patient while they are in ICU than in Western countries [10,20,22,23,25,26]. In contrast, family members from Western countries, while not having this involvement, expressed both a desire to be and that they did not want to be detached from caregiving [10,20,22]. Families also describe being advocates for the person, having a unique understanding of them and desire to let the clinicians know who they are, distinct from being a patient. Previous research on family members’ ability to recognize expressions of pain in TBI patients also shows that families believe they are best placed to advocate as they understand the personality and expressions of the patient [28].

There is undoubtedly a common thread of family members recognizing their role as changing from spouse, parent, or sibling to the role of a support person in this transitional phase in the ICU. Whiffin et al. found that in the post-acute phase, families describe losing their sense of self and time for themselves and becoming absorbed into the caregiving role [5]. Families can struggle to move past the injuries and the effects of the injury, fearing for the future of an injured person and their quality of life [5].

An enormous adjustment of the structure and functional roles awaits family members of patients with severe TBIs. Culturally in Western ICUs, personal care is predominantly given by clinical staff in the ICU. Most of the participants in the studies were female, apart from one study in India [21]. This difference may be explained by local cultural expectations stated in the study that male patients will be accompanied by a male family member [21]. The gender of study participants appears to be influenced by the gender of the patients with a TBI, who are predominantly male in all studies, in keeping with an international trend in TBI prevalence [4,29]. Further investigation may be needed to understand cultural influences on the burden of care. Further investigation may also be needed to ascertain if support and guidance may be given while a person is in the clinical environment to identify the future caregiver, and secondly to understand if supporting greater involvement while in ICU can help to bridge this transition from being a spouse, partner, parent, or sibling to the caregiver. Female participants in the studies reviewed recognize themselves to be future caregivers but also recognize a loss of their lives as they have known it. Warren et al. highlights female members of the family of patients with TBIs as being at a high risk of psychological morbidity post-intensive care [2]. Urgent research is warranted to understand what can be performed to assess and mitigate this risk while patients are in the acute care setting, to both safeguard the future care of TBI patients, but moreover safeguard the psychological well-being of families and future caregivers. Authors should discuss the results and how they can be interpreted from the perspective of previous studies and of the working hypotheses. The findings and their implications should be discussed in the broadest context possible. Future research directions may also be highlighted.

### Limitations

This review specifically considered family members of adult patients with TBI. Among TBI patient families, the psychological strain experienced may be directly related to injury severity. Therefore, family members of patients with severe TBI were only considered in this review. However, a large number of studies were excluded in which the injury was moderate to severe. There is an overall shortage of data available about family members’ experiences specific to the severe TBI patient group in the ICU. In addition, this review is restricted to a concentrated stage of the patient pathway that can last only days or weeks in the acute care journey. Thus, the onward patient journey and respective family experiences are not expressed in this review, nor are the sequelae of the ICU experiences. This is a suggested step in the prior literature [30]. Another consideration is that this review includes studies from very few countries and is restricted to English-language publications, resulting in limited generalizability of the global experiences of families. Finally, implicit bias may have been introduced in the findings of this review by virtue of the subject interest in family experiences in the ICUs represented in the retained papers.

## 5. Conclusions

This scoping review sought to explore the experiences of family members when a patient is in the ICU with a severe TBI. Rigorous and transparent methodology underpinned by Arksey and O’Malley [11] and JBI frameworks revealed three overarching themes with five subthemes. These results will offer richness and depth of understanding to clinicians regarding the experiences of families during this traumatic time. This review will also aid the direction of targeted interventions aimed at supporting family members while in the ICU.

### Recommendations or Implications for Further Research and Practice

The current scoping review highlights that family members need consistent communication and transparency at a time of extreme uncertainty. Further qualitative work to explore communication uncertainties with families at a time of high stress is recommended. To improve the therapeutic relationship between family members and clinical staff, this review recommends that healthcare professionals are forthcoming when communicating clinical information in the ICU and make this information readily available. In addition, further research with family members would help to understand how best to involve family members in patient care in an ICU environment. This review also highlights that family members, and future carers, are potentially at risk of psychological morbidity. Further research is needed into how to assess this risk.

## Figures and Tables

**Table 1:** Inclusion and exclusion criteria.

	Inclusion Criteria	Exclusion Criteria
Title and abstract	Population: Families of adults aged 18 or above with a severe TBI Primary and empirical research with qualitative and quantitative methodologies Written or translated into English Context Adult ICU setting, Non-acute, long-term, and rehabilitation research papers may be included where the context refers to the ICU setting Concept The experiences of family members	Infant, neonatal, pediatric, and children ICU (aged < 18 years) Reviews and opinion articles Non-English language papers
Full-text	Concept Studies that included family members who are described by other terminologies, i.e., caregivers, surrogate decision-makers	No full text is available Not peer-reviewed Studies that relate to the donor pathway only.

**Table 2:** Study characteristics.

Citation Details	Country	Study Design/Method	Participant Details	Population	Concept	Context
[10] Keenan and Joseph (2010)	Canada	Qualitative semi-structured interviews/Interviews	Mother—9 Father—3 Wife—5 Sister—4 Girlfriend—3 Brother—1	Twenty-five family members of 15 patients were admitted to the ICU with severe TBI.	Family member’s experience	Timepoint 1—within four days of ICU discharge. Timepoint 2—at discharge from an acute hospital. (Data taken only from time point 1)
[20] Bond et al. (2003)	USA	Exploratory qualitative descriptive design/Longitudinal qualitative interview	Mothers—2 Daughter—1 Father—1 Grandmother—1 Sister—1 Uncle—1	Aged above 18, severe TBI (GCS score < 8) in ICU	Needs of patients’ families	ICU for at least 24 h
[21] Kanmani et al. (2019)	India	Descriptive research design/Interviews to administer the quantitative questionnaire	Spouse—9 Daughter/son—17 Parents—24 Secondary relatives—6 Siblings—4	no age, primary caregivers providing care to a relative with TBI in ICU for more than one week (mild, moderate, and severe)	caregiver distress and burden	ICU for four days
[22] Lefebvre et al. (2005)	Canada	Qualitative retrospective study—secondary analysis/Qualitative Interviews	8 TBI patients & Family members (not specified), physicians, and health professionals	Family members of a patient with TBI—75% severe, 25% moderate	Experiences of individuals who sustained TBI, their families, and the physicians & professionals	Critical care onwards
[23] Lefebvre & Levert (2006)	Canada	Qualitative retrospective/Qualitative interviews	8 TBI patients & Family members (not specified), physicians, and health professionals	Family members of a patient with TBI—75% severe, 25% moderate	Experiences of individuals who sustained TBI, their families, and the physicians & professionals	Retrospective recall from experiences in ICU
[24] Piyakong (2014)	Thailand	Exploratory qualitative descriptive design/Qualitative interview	Wives—4 Mothers—2 Fathers—2 Niece—1	Family members of TBI patients; neurological critical care setting (ICU)	Exploring challenges and approaches for resolving challenges	ICU
[25] Kean (2010)	Scotland	Constructivist grounded theory/Qualitative interviews	Partner/spouse—1 Mother—3 Father—3 Sister—5 Brother—3	Family members of patients with TBI, ICU	Families experience ambiguous loss	ICU
[26] Quinn et al. (2017)	USA	Qualitative retrospective interviews/Qualitative interviews	Surrogates—16, relationship to TBI patient not stated	Aged 18 or over, primary decision maker for critically ill TBI patient within the last two years (extrapolating that critically ill in level 1 center = severe TBI)	Communication preferences	Level 1 trauma center (neuroICU)
[27] Jones et al. (2021)	USA	Secondary analysis of semi-structured interviews/Qualitative interviews	Surrogates—16, relationship to TBI patient not stated	Aged 18 or over, primary decision maker for critically ill TBI patient within the last two years (extrapolating that critically ill in level 1 center = severe TBI)	Communication of uncertainty	Level 1 trauma center (neuroICU)

Key: USA—United States of America; NeuroICU—Neurosurgical Intensive Care Unit.

**Table 3:** Study findings.

Citation Details	Aim of Study	Method of Analysis	Themes	Outcome Measure (Description/Validated)	Findings
[10] Keenan and Joseph (2010)	To identify the needs of individual family members of a relative who sustained a severe TBI and to determine if these needs changed over time.	Thematic analysis	(1) Getting the news (2) Uncertainty (3) Making sense of the news (4) Moving on	n/a	Getting the news—vivid memories and intense emotional reactions. Uncertainty—the uncertainty of survival, uncertainty in waiting, and uncertainty of prognosis “he was so critical, like it was hour by hour... that was the hardest... the waiting and the unknown”. Making sense of the news—seeking information from the clinical team and “intense need to know”. Moving on—the shift of focus of how to manage life outside the longer term. The needs of the family—Involvement in Care; Looking for progress; managing life; Holding on to hope; Information, Responding to the family’s needs—Professional support; Community support.
[20] Bond et al. (2003)	To explore the needs of patients’ families through individual interviews during the course of the patient’s stay in the ICU	Content analysis	(1) Need to know (2) Need for consistent information (3) Need for involvement in care (4) Need to make sense of the experience	n/a	(1) Families expressing the need for direct contact with the medical team, desire to know the truth, “Please give me some reality” (2) “Unable to know what is real and what is an opinion”. Receiving conflicting information from different physicians (3) Frustration with being left out of patient care—wish to be trusted with care tasks, feeling of being useless in patient care. (4) Wanting more detail about why things are carried out. Frightening experience that leads to relying on faith.
[21] Kanmani et al. (2019)	To assess the family burden and psychological distress among TBI caregivers at the emergency ICU	Descriptive statistics & independent burden *t*-test	n/a	Family burden and depression, anxiety, and stress scale (DAS-21).	The severity of TBI injury was associated with caregiver burden. The severity of TBI was likely to increase the burden on caregivers at ICU. Family burden score—44.7% moderate burden, 55.3% severe burden Statistically significant higher scores are reported in caregivers’ depression, anxiety, and stress levels from severe TBI in comparison to mild/moderate injuries.
[22] Lefebvre et al. (2005)	To investigate the experiences of individuals who had sustained a TBI, their families, the physicians, and health professionals involved from the beginning of acute care to their reintegration into daily life.	Content analysis	(1) Unclear communication (2) Feelings of uncertainty (3) Desiring transparency of information (4) Feelings of insignificance in patient care.	Internal validity	Families feel that the information being given to them by professionals regarding prognosis is inadequate, lending to significant uncertainty. They appear to also need support in that uncertainty. Families want to contribute to the care of the individual and feel a responsibility to let the clinical team know ‘who they are’ before the TBI. They experience feeling brushed aside and a lack of recognition for their role in patient care.
[23] Lefebvre & Levert (2006)	To investigate the experiences of individuals who had sustained a TBI, their families, the physicians, and health professionals involved in critical care episodes and subsequent rehabilitation.	Content analysis	(1) Shock (2) Lack of information (3) Uncertainty	Internal validity	There is a dividing line between before and after the TBI. There is a lack of information given to families, which restricts their ability to absorb what has happened. This contributes to the difficulty they face. Families sense the uncertainty from health professionals, and this vagueness contributes to their adjustment process from before to after.
[24] Piyakong (2014)	To explore challenges and approaches for resolving challenges that Thai family members face when engaging with their loved one with severe traumatic brain injury in the critical care setting	Content analysis	(1) Facing the uncertainty of a loved one’s illness (2) Dealing with personal suffering (3) Changing everyday life patterns.	n/a	Thai family members face the health challenges of uncertainty when a loved one suffers unconsciousness from TBI. Their approaches to managing challenges include the use of familiar resources to connect with the loved one and improve consciousness.
[25] Kean (2010)	To explore the families’ experiences with critical illness in the ICU and nurses’ perceptions of families	Ground theory—Constant comparative method	(1) Family experiences—Clinical uncertainty; functional uncertainty (2) Children—Adult’s power of controlling information; children’s agency; fishing for information	n/a	Core experiences—(1) clinical and functional uncertainty—(2) ambiguous loss (physically present, psychologically absent) embedded in (1). (3) Mapping the future; the impact of ambiguous loss on everyday family life embedded in (2)
[26] Quinn et al. (2017)	To explore key communication preferences and practices by stakeholders (surrogates and physicians) for the outcome prognostication during goals of care discussions for ciTBI	Thematic analysis	(1) Uncertainty and frustration regarding decision making (2) Seeking honest communication to inform decisions (3) Inconsistency of support in decision making (4) Lack of recognition of distress.	n/a	A majority of surrogate decision-makers felt unprepared in the decision-making process and struggled for many months afterward. Providing numeric prognostic estimates was helpful in this decision-making process. Families felt a sense of hope was important but needed the facts from physicians and not false hope. Emotion distress was caused by inconsistency in communication between physicians and the clinical team.
[26] Jones et al. (2021)	To identify strategies used by physicians specifically to communicate uncertainty as well as surrogates’ perceptions of this communication and of uncertainty.	Secondary Analysis using thematic analysis	(1) Ambiguity in goals of care (2) Prognostic uncertainty impacting decision making (3) Worry about making the ‘wrong decision.’	n/a	Practical decision-making is impacted by feelings of prognostic uncertainty. Too much ambiguity lends to a need for certainty, while too little ambiguity leaves families uncertain about whether hope should be ‘taken away’. Positive communication experiences are described as having questions answered, feeling included, and simple language is used.

Key: ciTBI—critically ill with TBI; n/a—Not Applicable.

## Data Availability

Not applicable.

## Supplementary Materials

The following supporting information can be downloaded at: https://www.mdpi.com/article/10.3390/jcm12134197/s1, Supplementary 1—PRISMA-ScR checklist; Supplementary 2—Search strategy; Supplementary 3—Excluded articles; Supplementary 4—Appraisals.
