# Supplementary material for: Exploring the Experiences of Family Members When a Patient Is Admitted to the ICU with a Severe Traumatic Brain Injury: A Scoping Review
Source: J Clin Med. 2023 Jun 21;12(13):4197. doi: 10.3390/jcm12134197 (PMC10342526; doi:10.3390/jcm12134197)
Supplement: Supplementary file 1 [file jcm-12-04197-s001.zip › Supplementary material 2 - search strategy.pdf]

Supplementary material 2

Search Strategies

OVID MEDLINE

Ovid MEDLINE(R) ALL <1946 to February 01, 2023>

Row	Search terms	Number of references
1	(ICU or "intensive care" or "critical care" or "critical* ill*" or ITU).mp.	317951
2	exp Critical Care/ or exp Intensive Care Units/	153670
3	exp Brain Injuries, Traumatic/	23005
4	("traumatic brain injury" or "traumatic brain injuries").mp.	47096
5	((head or crani* or cerebr* or capitis or brain* or forebrain* or skull* or hemispher* or "intra-cran*" or "inter-cran*") adj3 (injur* or trauma* or damag* or wound* or fracture* or contusion*)).mp.	217068
6	((head or crani* or cerebr* or brain* or "intra-cran*" or "inter-cran*") adj3 (haematoma* or hematoma* or haemorrhag* or hemorrhag* or bleed* or pressure)).mp.	106754
7	(Glasgow adj3 (coma or outcome) adj3 (scale* or score*)).mp.	23090
8	"rancho los amigos scale".mp.	45
9	("diffuse axonal injury" or "diffuse axonal injuries").mp.	1793
10	((brain or cerebral or intracranial) adj3 (oedema or edema or swell*)).mp.	26288
11	((unconscious* or coma* or concuss* or "persistent vegetative state") adj3 (injur* or trauma* or damag* or wound* or fracture*)).mp.	5299
12	exp Family/	362325
13	exp Caregivers/ or exp Caregiver Burden/	48647
14	(mother* or father* or mum* or dad* or parent or parents).mp.	887809
15	(family or families).mp.	1312407
16	(grandparent* or grandmother* or grandfather* or grandma* or grandpa* or sibling* or brother* or sister* or husband* or wife* or wives* or spous* or aunt or aunts or uncle or uncles or cousin*).mp.	198485
17	exp Parents/	138177
18	Grandparents/ or Siblings/	13786
19	1 or 2	324707
20	or/3-11	330374
21	or/12-18	2219170
22	19 and 20 and 21	913
23	exp Animals/ not Humans/	5088468

24	22 not 23	912
25	(exp Child/ or exp Infant/ or child*.mp. or infant*.mp. or baby.mp. or babies.mp. or NICU.mp. or neonat*.mp. or paediatric*.mp. or pediatric*.mp. or PICU.mp.) not exp Adult/	2490047
26	24 not 25	554
27	limit 26 to english language	495
