# Supplementary material for: Exploring the Experiences of Family Members When a Patient Is Admitted to the ICU with a Severe Traumatic Brain Injury: A Scoping Review
Source: J Clin Med. 2023 Jun 21;12(13):4197. doi: 10.3390/jcm12134197 (PMC10342526; doi:10.3390/jcm12134197)
Supplement: Supplementary file 1 [file jcm-12-04197-s001.zip › Supplementary material 3 - excluded articles.pdf]

Supplementary material 3 – Reasons for exclusion

**Reference**	**Reason for exclusion**
Aboubakr M, Yousaf MIK, Alameda G. Brain Death Criteria. 2021 Dec 27. In: StatPearls [Internet]. Treasure Island (FL): StatPearls Publishing; 2022 Jan–. PMID: 31424728.	No family experience
Bader, M.K. (2004). Changing Team Practice: Applying Evidence-Based Brain Injury Guidelines to Clinical Practice. Worldviews on Evidence-Based Nursing. 1(4), pp. 277	Conference abstract
Bogoch II, Sockalingam S, Bollegala N, Baker A, Bhalerao S. Family types in the neurotrauma intensive care unit. Am J Crit Care. 2005 Jul;14(4):283-4. PMID: 15980418.	Opinion article
Creutzfeldt CJ, Schutz REC, Zahuranec DB, Lutz BJ, Curtis JR, Engelberg RA. Family Presence for Patients with Severe Acute Brain Injury and the Influence of the COVID-19 Pandemic. J Palliat Med. 2021 May;24(5):743-746. doi: 10.1089/jpm.2020.0520. Epub 2020 Nov 17. PMID: 33210984; PMCID: PMC8064964.	Not severe TBI
Choustikova J, Turunen H, Tuominen SH, Coco K. Traumatic brain injury patients' family members' evaluations of the social support provided by healthcare professionals in acute care hospitals. Journal of Clinical Nursing. 2020;29(17/18):3325-3335. doi:10.1111/jocn.15359	Unable to determine TBI severity
Engli M, Kirsivali-Farmer K.Engli M, et al. Needs of family members of critically ill patients with and without acute brain injury.J Neurosci Nurs. 1993 Apr;25(2):78-85. doi: 10.1097/01376517-199304000-00003.J Neurosci Nurs. 1993. PMID: 8478558	Unable to determine TBI severity
Hoewing, B. (2017). Assessing the Quality of Education and Information Delivery to Family Members of Patients with Moderate to Severe Traumatic Brain Injury. https://repository.arizona.edu/handle/10150/624571	Quality improvement project
Hwang DY. Caring for Patients' Families (or Lack of Family) in Neurocritical Care. Neurocrit Care. 2017 Oct;27(2):151-153. doi: 10.1007/s12028-017-0470-z. PMID: 28916970.	Editorial
Hwang, D. Y., Knies, A. K., Mampre, D., Kolenikov, S., Schalk, M., Hammer, H., White, D. B., Holloway, R. G., Sheth, K. N., & Fraenkel, L. (2020). Concerns of surrogate decision makers for patients with acute brain injury: A US population survey. *Neurology, 94*(19), e2054-e2068. https://doi.org/10.1212/WNL.0000000000009406	No family experience
Johnson BP. One family's experience with head injury: a phenomenological study. J Neurosci Nurs. 1995 Apr;27(2):113-8. doi: 10.1097/01376517-199504000-00010. PMID: 7622948.	Under 18
Kreitzer, N., Bakas, T., Kurowski, B., Lindsell, C. J., Ferioli, S., Foreman, B., Ngwenya, L. B., Thomas, S., Keegan, S., Adeoye, O. (2020). The experience of caregivers following a moderate to severe traumatic brain injury requiring icu admission. Journal of Head Trauma Rehabilitation, 35, E299-E309. https://dx.doi.org/10.1097/HTR.0000000000000525	Unable to separate severe TBI in results
Lester EG, Mace RA, Bannon SM, Popok PJ, Gates MV, Meyers E, Tehan T, Sagueiro D, Rosand J, Macklin EA, Vranceanu AM. Can a Dyadic Resiliency Program Improve Quality of Life in Cognitively Intact Dyads of Neuro-ICU Survivors and Informal Caregivers? Results from a Pilot	No family experience

RCT. Neurocrit Care. 2021 Dec;35(3):756-766. doi: 10.1007/s12028-021-01222-3. Epub 2021 Apr 21. PMID: 33880701.	
Lin A, Vranceanu AM, Guanci M, Salgueiro D, Rosand J, Zale EL. Gender Differences in Longitudinal Associations Between Intimate Care, Resiliency, and Depression Among Informal Caregivers of Patients Surviving the Neuroscience Intensive Care Unit. Neurocrit Care. 2020;32(2):512-521. doi:10.1007/s12028-019-00772-x, 10.1007/s12028-019-00772-x	Unable to separate severe TBI in results
Lou W, Granstein JH, Wabl R, Singh A, Wahlster S, Creutzfeldt CJ. Taking a Chance to Recover: Families Look Back on the Decision to Pursue Tracheostomy After Severe Acute Brain Injury. Neurocrit Care. 2022 Apr;36(2):504-510. doi: 10.1007/s12028-021-01335-9. Epub 2021 Sep 2. PMID: 34476722; PMCID: PMC8412876.	Unable to separate severe TBI in results
Marks JP, Daggett LM. A critical pathway for meeting the needs of families of patients with severe traumatic brain injury. J Neurosci Nurs. 2006 Apr;38(2):84-9. doi: 10.1097/01376517-200604000-00003. PMID: 16681288.	Quality improvement project
Meurer K., Kubota R., Baskaran N., et al Stressors Experienced by Primary Caregivers of Severe Acute Brain Injury Patients Recovering from Coma in Neurointensive Care Units: Preliminary Findings from a Multicenter Qualitative Study. Neurology 2022;98(18 SUPPL):no pagination.	Conference abstract
Meyers E, Lin A, Lester E, Shaffer K, Rosand J, Vranceanu AM. Baseline resilience and depression symptoms predict trajectory of depression in dyads of patients and their informal caregivers following discharge from the Neuro-ICU. Gen Hosp Psychiatry. 2020 Jan-Feb;62:87-92. doi: 10.1016/j.genhosppsych.2019.12.003.	Unable to separate severe TBI in results
Meyers EE, Shaffer KM, Gates M, Lin A, Rosand J, Vranceanu AM. Baseline Resilience and Posttraumatic Symptoms in Dyads of Neurocritical Patients and Their Informal Caregivers: A Prospective Dyadic Analysis. Psychosomatics. 2020 Mar-Apr;61(2):135-144. doi: 10.1016/j.psym.2019.11.007.	Unable to determine TBI severity
Mohamed WRA, Leach MJ, Reda NA, Abd-Ellatif MM, Mohammed MA, Abd-Elaziz MA. The effectiveness of clinical pathway-directed care on hospitalisation-related outcomes in patients with severe traumatic brain injury: A quasi-experimental study. J Clin Nurs. 2018 Mar;27(5-6):e820-e832. doi: 10.1111/jocn.14194. Epub 2018 Jan 23. PMID: 29193516.	No family experience
Muehlschlegel S, Hwang DY, Flahive J, Quinn T, Lee C, Moskowitz J, Goostrey K, Jones K, Pach JJ, Knies AK, Shutter L, Goldberg R, Mazor KM. Goals-of-care decision aid for critically ill patients with TBI: Development and feasibility testing. Neurology. 2020 Jul 14;95(2):e179-e193. doi: 10.1212/WNL.0000000000009770. Epub 2020 Jun 17. PMID: 32554766; PMCID: PMC7455326.	No family experience
Norup, A., Snipes, D. J., Siert, L., Mortensen, E. L., Perrin, P. B., & Arango-Lasprilla, J. C. (2013). Longitudinal Trajectories of Health Related Quality of Life in Danish Family Members of Individuals with Severe Brain Injury. *The Australian Journal of Rehabilitation Counselling*, *19*(2), 71-83. https://doi.org/10.1017/jrc.2013.12	No family experience
O'Callahan, Julianne G. RN MD; Fink, Carol RN; Pitts, Lawrence H. MD; Luce, John M. MD FCCM [Miscellaneous] *Critical Care Medicine.* 23(9):1567-1575, September 1995.	No family experience

Oyesanya TO, Harris Walker G, Loflin C, Prvu Bettger J. Negotiating the transition from acute hospital care to home: perspectives of patients with traumatic brain injury, caregivers and healthcare providers. Journal of Integrated Care. 2021;29(4):414-424. doi:10.1108/JICA-04-2021-0023	Not ICU
Schutz RE, Coats HL, Engelberg RA, Curtis JR, Creutzfeldt CJ. Is There Hope? Is She There? How Families and Clinicians Experience Severe Acute Brain Injury. J Palliat Med. 2017 Feb;20(2):170-176. doi: 10.1089/jpm.2016.0286. Epub 2016 Oct 20. PMID: 27763820	Unable to separate severe TBI in results
Suppes A, Fins JJ. Surrogate expectations in severe brain injury. Brain Inj. 2013;27(10):1141-7. doi: 10.3109/02699052.2013.804201. Epub 2013 Jul 29. PMID: 23895513; PMCID: PMC5454804.	No family experience
Tate RL, Lane-Brown AT, Myles BM, Cameron ID. A longitudinal study of support needs after severe traumatic brain injury. Brain Inj. 2020 Jul 2;34(8):991-1000. doi: 10.1080/02699052.2020.1764101. Epub 2020 Jun 24. PMID: 32579407.	Not ICU
Trevick SA, Lord AS. Post-traumatic Stress Disorder and Complicated Grief are Common in Caregivers of Neuro-ICU Patients. Neurocrit Care. 2017 Jun;26(3):436-443. doi: 10.1007/s12028-016-0372-5. PMID: 28054288.	Not TBI
Verhaeghe ST, van Zuuren FJ, Defloor T, Duijnstee MS, Grypdonck MH. How does information influence hope in family members of traumatic coma patients in intensive care unit? J Clin Nurs. 2007 Aug;16(8):1488-97. doi: 10.1111/j.1365-2702.2006.01807.x. PMID: 17655537.	Not TBI
Verhaeghe ST, van Zuuren FJ, Defloor T, Duijnstee MS, Grypdonck MH. The process and the meaning of hope for family members of traumatic coma patients in intensive care. Qual Health Res. 2007 Jul;17(6):730-43. doi: 10.1177/1049732307303242. PMID: 17582017.	Not TBI
Voumard RR, Dugger KM, Kiker WA, Barber J, Borasio GD, Curtis JR, Jox RJ, Creutzfeldt CJ. Goal-Concordant Care After Severe Acute Brain Injury. Front Neurol. 2021 Sep 17;12:710783. doi: 10.3389/fneur.2021.710783. PMID: 34603183; PMCID: PMC8485029.	No family experience
Voumard R.R., Kiker W., Dugger K., et al A new normal after severe acute brain injury: An observational cohort using a sequential explanatory design. Ann. Neurol. 2020;88(SUPPL 25):S206. doi:10.1002/ana.25865	No family experience
Vranceanu AM, Bannon S, Mace R, et al. Feasibility and Efficacy of a Resiliency Intervention for the Prevention of Chronic Emotional Distress Among Survivor-Caregiver Dyads Admitted to the Neuroscience Intensive Care Unit: A Randomized Clinical Trial. JAMA netw. open. 2020;3(10):e2020807. doi:10.1001/jamanetworkopen.2020.20807, 10.1001/jamanetworkopen.2020.20807	Unable to separate severe TBI in results
Warren, A. M., Rainey, E. E., Weddle, R. J., Bennett, M., Roden-Foreman, K., Foreman, M. L. (2016). The intensive care unit experience: Psychological impact on family members of patients with and without traumatic brain injury. *Rehabil Psychol*. 61(2):179-85.	Unable to separate severe TBI in results
Yeager S, Doust C, Epting S, Iannantuono B, Indian C, Lenhart B, Manche D, Morris M, Newton B, Ortman L, Young K, Thomas K. Embrace Hope: an end-of-life intervention to	End of life only

support neurological critical care patients and their families. Crit Care Nurse. 2010 Feb;30(1):47-58; quiz 59. doi: 10.4037/ccn2010235. PMID: 20124020.	
