# Supplementary material for: Exploring the Experiences of Family Members When a Patient Is Admitted to the ICU with a Severe Traumatic Brain Injury: A Scoping Review
Source: J Clin Med. 2023 Jun 21;12(13):4197. doi: 10.3390/jcm12134197 (PMC10342526; doi:10.3390/jcm12134197)
Supplement: Supplementary file 1 [file jcm-12-04197-s001.zip › Supplementary material 4 - Appraisal.pdf]

Supplementary material 4 - Critical appraisal of selected studies

Qualitative research *	Section A - Are the results of the study valid?								Section B - Is it worth continuing?			Section C - Will the results help locally?			Appraisal
**Citation details**	1. Was there a clear statement of the aims of the research?	2. Is a qualitative methodology appropriate?	3. Was the research design appropriate to address the aims of the research?	4. Was the recruitment strategy appropriate to the aims of the research?	5. Was the data collected in a way that addressed the research issue?	6. Has the relationship between researcher and participants been adequately considered?			7. Have ethical issues been taken into consideration?	8. Was the data analysis sufficiently rigorous?	9. Is there a clear statement of findings?	10. How valuable is the research?			
10. Keenan and Joseph (2010)	Yes	Yes	Yes	Can't tell	Yes	No			No	Yes	Yes	Contribution related to other evidence and limitations identified. Questionable value to future research, not evident. Difficult readability.			Medium
20. Bond et al (2003)	Yes	Yes	Yes	Yes	Yes	Yes			Yes	Yes	Yes	Contribution related to other evidence, new areas of research and limitations identified. Valuable relevance to practice.			High
22. Lefebvre et al (2005)	Yes	Yes	Yes	Yes	Yes	No			No	Yes	No	Contribution related to other evidence, new areas of research and limitations identified. Difficult readability; findings extracted by KH.			Low
23. Lefebvre & Levert (2006)	No	Can't tell	Can't tell	Can't tell	Can't tell	No			No	Yes	No	Contribution related to other evidence, new areas of research not identified, limitations identified. Difficult readability;			Low

												findings extracted by KH.			
24. Piyakong (2014)	Yes	Yes	Yes	Can't tell	Can't tell	No			No	No	Yes	Contribution not related to other evidence, limitations not identified. Questionable value to future research, not evident. Pilot study.			Low
25. Kean (2010)	Yes	Yes	Yes	Yes	Yes	Can't tell			Yes	Yes	Yes	Contribution related to other evidence, new areas of research and limitations identified. Valuable novel insight, relevant to practice.			High
26. Quinn et al (2017)	Yes	Yes	Yes	Yes	Yes	Yes			Yes	Yes	Yes	Contribution related to other evidence, new areas of research and limitations identified. Valuable for development of decision aid. Difficult readability.			Medium
27. Jones et al (2021)	Yes	Yes	Yes	Yes	Yes	Can't tell			Yes	Yes	Yes	Contribution related to other evidence, new areas of research and limitations identified. Valuable for development of decision aid. Difficult readability.			Medium
**Cohort study****	**Section A - Are the results of the study valid?**								**Section B - Is it worth continuing?**			**Section C - Will the results help locally?**			
**Citation details**	1. Did the study address a clearly focused issue?	2. Was the cohort recruited in an acceptable way?	3. Was the exposure accurately measured to minimise bias?	4. Was the outcome accurately measured to minimise bias?	5. (a) Have the authors identified all important confou	5. (b) Have they taken account of the confounding factors	6. (a) Was the follow up of subjects	6. (b) Was the follow up of subjects long	7. What are the results of the study?	8. How precise are the results?	9. Do you believe the results?	10. Can the results be applied to the local population?	11. Do the results of this study fit with	12. What are the implications of this	

					nding factors?	in the design and/ or analysis ?	complete enough?	enough?					other available evidence ?	study for practice?	
21. Kanmani et al (2019)	Yes	Yes	Yes	Yes	Yes	Yes	No	Can't tell	Carers of TBI patients reported financial burden and anxiety	Fair (mean and StDev presented)	Yes	No	Yes	Can't tell	Medium

*Critical Appraisal Skills Programme (2021). CASP Qualitative Checklist. [accessed 24/04/2023]

**Critical Appraisal Skills Programme (2021). CASP Cohort study Checklist. [accessed 24/04/2023]
